# Towards Fast Plume Source Estimation with a Mobile Robot

**DOI:** 10.3390/s20247025

**Published:** 2020-12-08

**Authors:** Hugo Magalhães, Rui Baptista, João Macedo, Lino Marques

**Affiliations:** 1 Institute of Systems and Robotics, Department of Electrical and Computer Engineering, University of Coimbra, 3030-290 Coimbra, Portugal; hugo.magalhaes@isr.uc.pt (H.M.); rui.baptista@isr.uc.pt (R.B.); jmacedo@isr.uc.pt (J.M.); 2 Centre for Informatics and Systems, Department of Informatics Engineering, University of Coimbra, 3030-290 Coimbra, Portugal

**Keywords:** mobile robotics, gas source localisation, particle filter

## Abstract

The estimation of the parameters of an odour source is of high relevance for multiple applications, but it can be a slow and error prone process. This work proposes a fast particle filter-based method for source term estimation with a mobile robot. Two strategies are implemented in order to reduce the computational cost of the filter and increase its accuracy: firstly, the sampling process is adapted by the mobile robot in order to optimise the quality of the data provided to the estimation process; secondly, the filter is initialised only after collecting preliminary data that allow limiting the solution space and use a shorter number of particles than it would be normally necessary. The method assumes a Gaussian plume model for odour dispersion. This models average odour concentrations, but the particle filter was proved adequate to fit instantaneous concentration measurements to that model, while the environment was being sampled. The method was validated in an obstacle free controlled wind tunnel and the validation results show its ability to quickly converge to accurate estimates of the plume’s parameters after a reduced number of plume crossings.

## 1. Introduction

The occurrence of natural catastrophes and human-caused chemical disasters may cause serious environmental impacts, creating the need for methods that quickly estimate the location of the release source, aiding the response teams. The problem of Source Term Estimation (STE) consists of using information from distributed sensors to estimate the parameters of a chemical source. This is often tackled with mathematical methods that assimilate data from static sensor networks [1] or from mobile sensing platforms [2]. The Gaussian Plume model is at the core of many regulatory atmospheric dispersion models and is typically used to model the dispersion of contaminants on the atmosphere [3]. It models the time-averaged chemical dispersion in an environment and thus it is often quite distinct from the instantaneous plumes that can be observed. This is due to the natural phenomena associated with air flow and chemical dispersion. The gas particles, once released, flow with the wind and spread through molecular diffusion and turbulent dispersion, creating intermittent and meandering gas distribution, which are called gas plumes. Probabilistic prediction and data assimilation methods have been extensively used to produce accurate or satisfactory estimates of odour sources. The Particle Filter, a Bayesian method also known as Sequential Monte Carlo, has been widely used in highly nonlinear applications with mobile robots. The idea is to generate a population of random particles, each one encoding a solution of the target problem, which are iteratively improved. These filters have also previously been used for odour source localisation, both for single and for multiple mobile robot systems [4,5,6,7,8]. However, due to the exponential growing need of the number of particles with the dimension of the state vector, the previous works have usually estimated a reduced number of source parameters. Additionally, these works did not validate or evaluate their results in controlled environments, making difficult to compare multiple experiments. Independently from the estimation process, any method can only perform well if it is fed with relevant data, which depends on the strategy employed to sample that data across the active region of the odour plume. Given the need for fast estimations, as well as the current autonomy limitations of mobile robots, efforts should be made to sample the environment in the most efficient manner. This paper contains three main contributions:

1. Two-stage estimation algorithm: Most of the existing approaches to STE that rely on Particle Filters do so by using short state vectors, i.e., estimate a reduced number of parameters of the underlying odour dispersion models. Conversely, the present work intends to estimate many parameters of the Gaussian Plume model. Traditionally, this would imply a substantial increase of the number of particles used, slowing down the estimation process. This work proposes an inverse method to provide rough estimates of the parameters of the Gaussian Plume model from a set of environmental measurements obtained from a single plume crossing. These rough estimates are used to restrict the search space of the Particle Filter, improving the quality and speed of the estimation process.
2. Bio-inspired navigation strategy: A navigation strategy is proposed to improve the speed of the estimation process and reduce the effort made by the robot. Drawing inspiration from counterturning behaviours present in nature, a Genetic Algorithm is applied to evolve the sequence of motions that optimise the quality of the environmental measurements.
3. Wind tunnel validation of the proposed approach: Thus far, most of the existing works test their approaches either in simulation or in uncontrolled environments. The present work evolves the sampling strategies in a realistic simulator and validates the complete approach in a controllable wind tunnel where distinct environmental conditions may be created.

The remaining of this paper is organised as follows: Section 2 presents related work on Source Term Estimation methods, Section 3 presents the problem formulation as well as the methods used for the estimation process; Section 4 describes the proposed solution. The experimental setup is presented in Section 5; Section 6 describes the experimental results obtained in the evaluation of the work; and Section 7 draws the conclusions from this work and provides insight into future endeavours.

## 2. Related Work

There are two different strands of work for locating odour sources: plume tracing methods and source estimation methods. The plume tracing methods aim to use environmental information to guide the robot to the location of the odour source. Conversely, source estimation methods focus on using environmental data to estimate the parameters of the chemical source, without the need to navigate to its location. This section presents some of the main works of both approaches.

### 2.1. Plume Tracing Methods

Plume tracing methods attempt to use environmental information to guide the robot to the location of the chemical source. The plume tracing methods have three well defined stages, each requiring a distinct behaviour: (1) Plume Search, where the robot must explore the environment, searching for the initial odour cues; (2) Plume Track, where the robot is in contact with the odour plume and must follow it to the vicinity of its source; and (3) Source Declaration, where the robot must pinpoint the location of the odour source.

Due to the ability of animals to successfully locate odour sources, most of the existing plume tracing methods are inspired by their behaviours, which are designed to work in specific conditions. One of the most important environmental conditions is the strength and stability of the air flow. In environments where the flow is weak or non-existent, biological organisms employ chemotactic strategies, which use information about the chemical gradient. One such behaviour is the biased random walk observed in bacteria and other small organisms [9]. In environments containing strong winds, animals typically take advantage of the airflow information to guide their search process [10]. A popular anemotactic approach is inspired by the behaviours of the male silkworm moth while tracking a trail of pheromone [11].

### 2.2. Source Term Estimation

The goal of Source Term Estimation (STE) is to predict the dispersion of chemical plumes by estimating the parameters of the emitting source, such as the location and emission rate. Usually, two main approaches are used to tackle this problem: optimisation methods and probabilistic approaches, both of which have the objective of finding the best match between the measured and predicted data. Regarding the probabilistic approaches, particle filter algorithms have been widely used in this field of research [4,5,6,7,8,12] due to their ability to deal with multimodal distributions and highly nonlinear applications. This method consists of generating a large number of random hypothesis (particles) to approximate the posterior probability of a state vector. One of the downsides of particle filters is their computational complexity, which is proportional to the number of particles and observations. Bourne et al. [7] employed a Particle Filter to estimate the plume source parameters on a multi-robot STE approach. The proposed method was validated in a small arena with a custom humidity generator and three mobile robots. Li et al. [4] proposed an odour source localisation algorithm based on a particle filter. The method employs an exploration behaviour that aims to improve the information gain when a target chemical is detected. The detections are binarized and the validation was done with a mobile robot in an open, uncontrolled, environment. Neumann et al. [6] proposed to solve the STE problem by combining a plume tracking algorithm and a particle filter. The plume tracking algorithm is used to reach the vicinity of the odour source, at which point the particle filter is employed for declaring the source. Binary gas information is used during the plume acquisition and plume tracking stages, whereas the concentration values are used on the source declaration step. The method is validated in simulation and outdoors with a micro-drone. Ristic et al. [12] proposed a particle filter with Rao–Blackwell dimension reduction in order to estimate the location and emission rate of a plume source. With this method, the posterior density of the source emission rate, conditioned by the source position is computed analytically, and the estimation is only performed on the position states of the model. Numerical tests with simulated and experimental data show the improvements of the method. Lu et al. [5] dealt with the problem of odour source localisation using multiple mobile robots. A particle filter was used to estimate the position of the odour source from the observations of the entire robot group. The source position is estimated based on wind information and chemical detection events. The proposed approach was evaluated in simulation, with the results showing that the search time was reduced by cooperation. Park et al. [8] propose a multi-robot particle filter for estimating the position of an odour source as well as its emission rate. A set of simulation-based experiments are conducted to study the impact of the number of robots as well as of their coordination mechanism. The results showed that using coordination between the robots increased the performance of the estimation method.

To sum up, the existing works focus mainly on estimating a reduced number of parameters of the chemical plume. Most works validated the proposed approach with simulations, and the few that used real world experiments did so in uncontrolled environments. Moreover, the existing works often rely on binary chemical detections, rather than using the actual chemical concentration values. The present work aims to go one step further, estimating a broader number of parameters. A two-step estimation method is proposed to cope with the increased problem complexity and optimized zig-zagging trajectories are used to devise the sampling trajectories that optimise the quality of the information gathered.

## 3. Methods

This section describes the methods used in the present work for addressing the STE problem.

### 3.1. Problem Statement

Consider a mobile robot moving in known positions x(t) across a $R^{2}$ space. The robot is equipped with a gas sensor and a 2D anemometer that enable acquiring the gas concentration c(x(t)) and wind speed u(x(t)) along its trajectory. Assume an open, but bounded workspace, with air flowing in a dominant direction $\bar{u}$, containing an odour source located in an unknown position $\left(x_{s},y_{s},h_{s}\right)$ and releasing a chemical vapour at constant, but unknown rate *Q*. It should be noted that the odour plume generated by the odour source in this environment will not be smooth, but it will contain internal intermittency, generated by the turbulence created by the interaction in between the air flowing and the surfaces of the environment. The problem here is estimating the location and intensity of the odour source based on measurements collected by the robot while it covers the environment. The efficiency of this estimation process will depend on the Source Term Estimation algorithm employed and from the quality of the data provided to that algorithm. The later depends on the trajectory of the robot while covering the environment.

### 3.2. Plume Model

A three-dimensional Gaussian Plume model, as described by Equation (1), describes time-averaged spatial distribution of a chemical vapour $\bar{c}\left(x,y,z\right)$ released from a point source located in position (0,0,h), in a referential centred on the source, and releasing the chemical at a rate *Q*. The environment is supposed to be homogeneous, unbounded, and flat, and the average downwind direction $\bar{u}$ is assumed to be aligned with the *x* coordinate axis. The crosswind direction is assumed to be aligned with the *y* coordinate axis and *z* represents the distance to the soil surface, in the previously mentioned referential [3]:

(1) $$\begin{array}{c} \bar{c}\left(x,y,z\right)=\frac{Q}{2\pi \bar{u}\sigma_{y}\sigma_{z}}\exp\left(\frac{-y^{2}}{2\sigma_{y}^{2}}\right)\left[\exp\left(\frac{-{\left(z-h\right)}^{2}}{2\sigma_{z}^{2}}\right)+\exp\left(\frac{-{\left(z+h\right)}^{2}}{2\sigma_{z}^{2}}\right)\right] \end{array}$$

The lateral and vertical dispersion coefficients, $\sigma_{y}$ and $\sigma_{z}$ respectively, are functions of the downwind distance to the source (*x*), frequently modelled by the polynomials of Equations (2) and (3), whose coefficients depend on the atmosphere stability [13]:

(2) $$\sigma_{y}=ax^{b}$$

(3) $$\sigma_{z}=cx^{d}$$

### 3.3. Particle Filter Algorithm

The objective of a Particle Filter is to recursively approximate the posterior probability density function (PDF) for the system states with a group of particles using a series of weighted random samples and observation values [14]. Particle Filters can be used to estimate non-Gaussian distributions and nonlinear processes representing belief by random samples. The downside is the higher computational power required for large state spaces, as the amount of particles that must be generated grows exponentially with the number of dimensions of the space.

Each of the particles is a hypothesis consisting of a state $x_{t}^{\left(i\right)}$ and a corresponding weight $w_{t}^{\left(i\right)}$ representing the likelihood of that particle (Equation (4)):

(4) $$\chi_{t}:=\left\{\left(x_{t}^{i},w_{t}^{i}\right)\right\}\;i\;\in \left\{1,\ldots ,N\right\}$$

The likelihood is obtained by evaluating how well each hypothesis matches the observations $y_{t}$, where a better match results in a more significant weight value (i.e., better fitness value). Depending on their weight, the particles have a higher or lower chance to be replicated to the next iteration through the resampling process, also known as sequential importance resampling (SIR). The purpose of this method is to replace hypothesis with lower weights by those with higher weights, in order to improve the convergence of algorithm. This process is iterated multiple times until an estimate of the state vector *x* given by Equation (5) is obtained. The posterior density function of state is approximated as given in Equation (6):

(5) $$x_{t}=\sum_{i=1}^{N}w_{t}^{i}x_{t}^{i}$$

(6) $$p\left(x_{t}|c_{1:t}\right)\approx \sum_{i=1}^{N}w^{i}\delta \left(x_{t}-x_{t}^{i}\right)$$

In an STE process, the Gaussian Plume model is combined with the Particle Filter through a state space composed by the state transition model and the measurement model presented by Equations (7) and (8):

(7) $$x_{t+1}=x_{t}+v_{t}$$

(8) $$c_{t}=c\left(x,y,t\right)$$

The variable x represents our hypothesis (i.e., a particle), each containing the parameters to be estimated. The $v_{t}$ follows a normal distribution $v_{t}∼N\left(0,\sigma_{v}\right)$ and is used to update the particles on the prediction step during the filter iterations. $c_{t}$ represents the measured concentration values on multiple positions of the odour plume.

## 4. Proposed Solution

**Figure 1:**
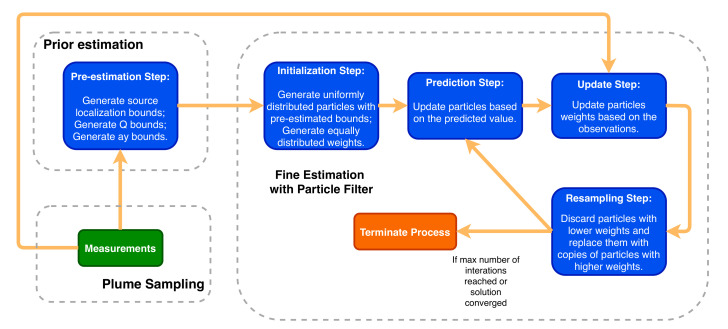
Overall strategy of the proposed method.

This work proposes to tackle the problem of STE by decomposing it into two sub-problems: (1) how to sample the environment and (2) how to use the information gathered to estimate the parameters of the chemical source. This section describes the proposed approaches for each sub-problem, starting with the trajectories made to sample the plume (Section 4.1) and moving on to how to estimate the parameters of the source (Section 4.2). Figure 1 presents the overall strategy of the the proposed method.

### 4.1. Sampling Strategy

Before estimating the parameters of the odour source, the robot must move in the environment, gathering samples of gas concentration and wind velocity.

**Figure 2:**
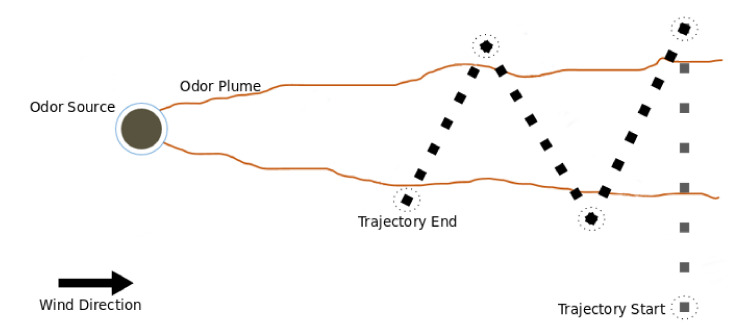
Example of an evolved trajectory. The grey dotted line represents the initial plume crossing, whereas the black dotted line shows the subsequent crossings, each with a different offset to the crosswind direction.

In nature, animals track scents with counterturning behaviours [10]. Two types of counterturning behaviours can be distinguished: (1) zigzagging, when the agent changes its position in the upwind axis; and (2) casting, when the motion is solely in the crosswind axis. In the zigzag behaviours, the angles of the counterturns have to be defined and do not have to necessarily be the same. In this work, the purpose of moving the robot is not to find the odour source, but to sample the environment in a way that optimises the quality of the estimation. Preliminary experiments showed that performing an initial plume crossing in the crosswind direction provided good data for the pre-estimation method, but was insufficient for accurately estimating the parameters of the chemical plume. To that end, and drawing inspiration from the upwind zigzag motions of the Dung Beetle [15], the trajectories were defined as consisting of two parts: (1) a plume searching stage and (2) a plume transversing stage. In the first stage, the robot moves directly crosswind (casting), halting after leaving the chemical plume. This stage is particularly important for the prior estimation step. In the second stage, the robot performs a series of diagonal motions to sample the environment (zigzagging). These motions are straight lines performed at an offset to the crosswind direction. As soon as the robot exits the plume, it will select the next offset, apply it to the opposite crosswind direction, and go back into it. An example trajectory is depicted in Figure 2. The question now is how to optimally select these offsets. In this work, a Genetic Algorithm shall be used to evolve the trajectories of the robot.

Evolutionary Algorithms [16] are a family of stochastic search heuristics loosely inspired by the principles of Natural Selection and Mendel’s genetics. Two of its sub-families are Genetic Algorithms (GAs) and Genetic Programming (GP). The main difference between them is that GAs evolve solutions for a given problem, whilst GPs evolve computer programs that, once executed, produce those solutions.

In this work, a GA is used to evolve the sampling trajectories. Each individual encodes a trajectory and, as previously mentioned, each trajectory is composed of two parts: an initial plume crossing directly in the crosswind direction and the motions to perform when sampling the plume. In turn, these motions consist of straight movements with an offset to the crosswind direction. Thus, the genotype of each individual consists of a vector of real-valued numbers, corresponding to the offsets to be added to the crosswind direction towards upwind on each plume crossing.

The size of each genotype is allowed to vary between 1 and 5 offsets. As a result, the trajectories consist of an initial plume crosswind with no offset to the crosswind direction, followed by a sequence of 1 to 5 diagonal plume crossings. The maximum length for the trajectory was selected as the results of preliminary experimentation showed no further performance gains with longer trajectories. Moreover, as moving the robot in the environment is a time and energy-consuming process, shorter trajectories are preferred and thus the GA is allowed to search for shorter trajectories that optimise the estimation quality.

The algorithm starts by creating a population of randomly-generated trajectories, which must be evaluated. The evaluation of each trajectory consists of running a simulation and collecting environmental data along the mentioned trajectory. In this work, the simulator proposed in [15] is used and each trajectory is evaluated until its termination or until the simulation time expires. The samples collected in simulation are fed into the particle filter, which outputs the best estimate for the source’s parameters. Analysing the results of preliminary experimentation, it was possible to verify that there are multiple sets of parameters for the Gaussian Plume model that fit the collected samples. i.e., it is possible to parametrise the Gaussian Plume model with the source at various locations if the remaining parameters are also modified so that it matches the measurements from the environment. As a result, and due to the various parameters having very different magnitudes, we opted by computing the fitness of each trajectory only as the error (Euclidean distance) between the estimation of the source’s position and the ground truth. After evaluation, the trajectories are evolved over a set of generations. On each generation, a subset of trajectories are chosen to act as mates, producing offspring through one point crossover and Gaussian mutation. The parameters of the GA and of the simulator are respectively presented in Table 1 and Table 2.

#### Evolution Results

**Figure 3:**
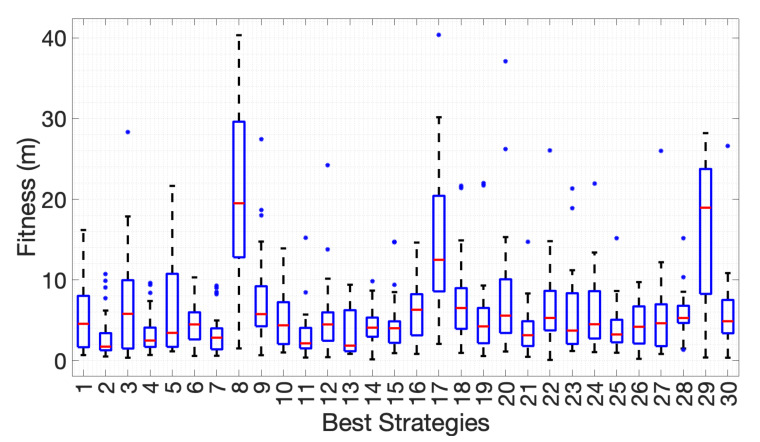
Fitness of the best strategies on the 30 instances of the simulation environment.

Thirty independent runs of the GA were made, yielding 30 strategies. In order to assess the robustness and true worth of the strategies, each one was run in the 30 instances of the simulation environment. Figure 3 presents the boxplots of the obtained fitness values (Euclidean distance between the estimated and real position of the odour source) for each of the best strategies. From the boxplots, it is clear that some strategies over-fit their environment, but have poor generalisation ability. In this work, the maximum acceptable error is considered to be of 5 m in the simulation environment and 0.5 m in the real world arena. Given the dynamic nature of the odour dispersion process, and the possible differences in the location of the robot and chemical source, it is not feasible to find an overall best set of angles. However, focusing on the strategies that attain a median error below 5 m, a pattern emerges, where, apart from the initial zero-offset crossing, a good quality strategy should have a sequence of at least four crossings with offsets between 12° and 45°.

### 4.2. Parameter Estimation

The parameters of the source are estimated with a particle filter. Each particle encodes the position and emission rate of the chemical source, along with estimates of parameters that are specific to the Gaussian plume model. In turn, the Gaussian plume model is used by the Particle Filter to evaluate the quality of each particle. This is done by comparing the environmental samples collected by the robot to the Gaussian plume model parametrised with each particle.

The high dimensionality of the estimation vector creates a large search space, where it is difficult for the Particle Filter to find good estimates. As a result, and based on the environmental data collected by the robot, a prior estimation method is proposed for bounding the search space for the location and emission rate of the odour source, as well as for the horizontal dispersion of the plume. This restriction of the search space optimises the generation of the particles, and consequently the search process of the Particle Filter. This section presents the proposed prior estimation method, as well as the particle filter algorithm.

#### 4.2.1. Prior Location Estimation

Considering that a set of environmental samples of a plume crossing is obtained, the coordinates of the location of the first $\left(x_{in},y_{in}\right)$ and last $\left(x_{out},y_{out}\right)$ odour detection can be used to narrow the region of the search space for particle generation. Due to the nature of the odour dispersion model, and considering a uniform wind along the *x* axis, the location of the plume’s source $\left(x_{s},y_{s}\right)$ is likely to be between $y_{in}$ and $y_{out}$ and on $x_{s}<\min\left(x_{in},x_{out}\right)$.

This region is considerably smaller than the entire arena and is an approximation of the real value. The initial particles will be generated within this region and, as a result of the reduced search space, fewer particles may be used, the initial standard deviation will be lower and the weight values will reflect the reduced error due to the larger proximity to the location of the source.

#### 4.2.2. Prior Emission Rate Estimation

According to the law of conservation of energy, the total energy of an isolated system remains constant over time. When crossing the plume with the robot, the concentration values are obtained, along with a displacement. Assuming a uniform air flow, the area under the concentration curve along the *y* coordinates will provide an approximation of the maximum concentration value measured near the source. This value can be used to bound the interval of the source’s emission rate. The area under the concentration curve is computed through its integral, as presented in Equation (9):

(9) $$Q=\int_{z_{min}}^{z_{max}}\int_{y_{min}}^{y_{max}}\bar{c}\left(y,z\right)\;\bar{u}\;d_{y}\;d_{z}$$

$\bar{c}\left(y,z\right)$ represents the gas concentrations measured by the sensor that equip the robot at each (x,y) location along the plume crossing; $\bar{u}$ is the mean wind velocity; $y_{min}$ and $y_{max}$ respectively correspond to the *y* coordinates where the robot entered and exited the plume. The prior estimation of the emission rate is obtained by converting the value resulting from computing this integral. This process allows for limiting the search space of the emission rate for the particle filter and optimising the generation of the initial particles.

#### 4.2.3. Prior Sigma Estimation

In a recent work [17], a method for computing the horizontal plume spread ($\sigma_{y}$) was proposed. This method is based on the crosswind integration of the concentration measurements, in order to determine the second moment of the mean of the data, as shown in Equation (10):

(10) $${\langle Y\rangle }^{2}=\frac{1}{A}\int_{-\infty }^{+\infty }{\left(Y-Y_{0}\right)}^{2}c\left(x,y\right)d_{y}$$

where *Y* is the cross-plume coordinate in meters, c(x,y) is the measured concentration, $Y_{0}$ is the weighted plume centreline, and *A* is the integrated concentration, computed through Equation (11):

(11) $$A=\int_{-\infty }^{+\infty }c(x,y)d_{y}$$

$\sigma_{y}$ is obtained as a square root of ${\langle Y\rangle }^{2}$. As the estimation process is computed on observations from plume crossings, the measured concentration data and the respective locations contain the information needed to apply this method to obtain a pre-estimation of $\sigma_{y}$. *Y* is obtained from the *y* location values of the trajectory of the robot. $Y_{0}$ is obtained from the position with the highest measured concentration value, approximately corresponding to the plume centreline. c(x,y) is the multiple chemical concentration measurements obtained along the trajectory of the plume crossing. From this estimation, a correlation between $\sigma_{y}$ and $a_{y}$ is obtained serving as a reference to generate the bounds $ay_{lb}$ and $ay_{ub}$ for this state on the Particle Filter.

#### 4.2.4. Particle Filter

This work uses a SIR Particle Filter, as the one described in the previous section, to estimate the parameters of an odour plume after each plume crossing. Let us consider the state vector $x=\{x_{s},y_{s},Q,a_{y},b\}$, containing the parameters of a 2D Gaussian plume, as described by Equation (12), where $x_{s}$ and $y_{s}$ represent the source location, *Q* the emission rate, and $a_{y}$ and *b* are the coefficients from the crosswind dispersion $\sigma_{y}=a_{y}{\left(x-x_{s}\right)}^{b}$:

(12) $$\begin{array}{c} \bar{c}\left(x,y\right)=\frac{Q}{2\pi \bar{u}\sigma_{y}}\exp\left(\frac{-y^{2}}{2\sigma_{y}^{2}}\right) \end{array}$$

Additionally, let us consider a set of *N* hypothesis, represented by $\chi_{t}:=\left\{\left(x_{t}^{i},w_{t}^{i}\right)\right\},i\in \left\{1,\ldots ,N\right\}$ particles, containing a state vector $x_{t}^{i}$ and its respective weight $w_{t}^{i}$, whose value represents a degree of confidence about how the state vector explains the data observed in the field. A Particle Filter uses the following steps to adjust iteratively the values of each particle, searching for sets of particles that better explain the observed data.

1. Filter initialisation: Generate a set of *N* particles $\chi_{0}t$ with initial values $x_{0}^{i}$ uniformly spread across a search space with boundaries defined by the previous estimation process (Equation (13)) and uniform weights $w_{0}^{i}=1/N$:
  (13) $$X^{i}=\left\{\begin{array}{c} x_{s}^{i}=U\left(x_{in},x_{out}\right)\\ y_{s}^{i}=U\left(y_{in},y_{out}\right)\\ Q^{i}=U\left(Q_{lb},Q_{ub}\right)\\ a_{y}^{i}=U\left(ay_{lb},ay_{ub}\right)\\ b^{i}=U\left(b_{lb},b_{ub}\right) \end{array}\right.$$
  The boundaries $(b_{lb}$ and $b_{ub}$) for parameter *b* were not pre-estimated, but this parameter is widely studied, and its range is well defined in the literature.
2. Prediction: Predict the next state of each particle following the equation $x_{t+1}=x_{t}+v_{t}$, where $v_{t}$ is an added random noise that follows a normal distribution $N(0,\sigma_{r})$. $\sigma_{r}$ is the defined variance of each parameter, defined to prevent a premature convergence of the particles and allow better exploration of the search space.
3. Update: All particles are evaluated against the measured concentration values using the following fitness function:
  (14) $$e_{t}^{1:s}=f\left(x_{t}^{i}\right)-y_{1:s},$$
  where *f* is the Gaussian plume model from Equation (12), $x_{t}^{i}$ the particle being evaluated, and $y_{1:s}$ the concentration measurements from the plume crossing with *s* the observation number. The weight of each particle is then updated according to Equation (15) and is later normalised with Equation (16), so all weights sum to 1:
  (15) $$w_{t}^{i}=\frac{1}{\sum_{s=1}^{n_{s}}{\left(e_{t}^{s}\right)}^{2}}$$
  (16) $$w_{t}^{i}=\frac{w_{t}^{i}}{\sum_{i=1}^{N}w_{t}^{i}}$$
4. Resampling: In the resampling process, the particles are selected with probability proportional to their weight, thus the best particles will appear more often in the next iteration and the worst particles will disappear in the process. The systematic resampling [18] is used when the effective number of particles ($N_{eff}$) is below a threshold, being $N_{eff}$ by Equation (17),
  (17) $$N_{eff}=\frac{1}{\sum_{i=1}^{N}w_{i}^{2}}$$
5. Compute estimations: The state estimation and the variance of the particles on each iteration is computed by Equations (18) and (19), respectively.
  (18) $$x_{t}=\sum_{i=1}^{N}w_{t}^{i}x_{t}^{i}$$
  (19) $$var_{t}=\sum_{i=1}^{N}w^{i}\left(x_{t}^{i}-x_{t}\right)$$
6. Check convergence: If the solution converges or a maximum number of iterations is reached, the filter terminates and outputs the last state estimation, variance, weights, and particles distribution. Otherwise, the process will repeat from the prediction step.

## 5. Experimental Setup

The main elements employed to validate the proposed methodology are a controllable testing environment and a mobile platform, able to estimate its pose and to measure the concentration of the target gas and the wind speed at its location.

### 5.1. Testing Environment

**Figure 4:**
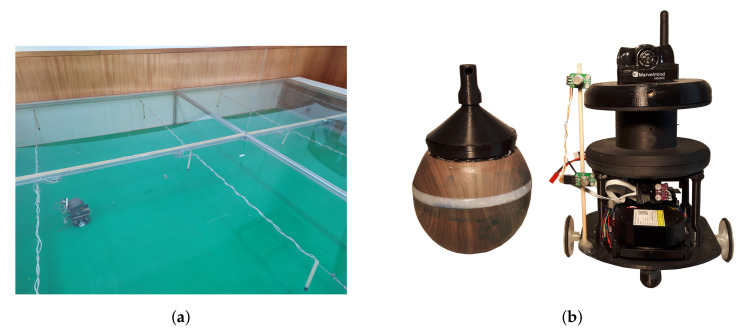
Experimental setup. (**a**) Wind tunnel environment; (**b**) odour source (left) and mobile robot (right).

The testing environment employed to validate the proposed methodology is a large wind tunnel-like environment, with 3 m width by 4 m long, by 0.5 m height, as already used in previous works (e.g., [19]). This environment is large enough to carry-out olfactory experiments with single or multiple small robots (see Figure 4a). The airflow inside the environment is controlled from 0 until 1 m/s through an array of 24 axial ventilators, whose speed can be individually controlled. This airflow is monitored with a WindSonic ultrasonic anemometer from Gill Instruments (Lymington, UK). The gas source employed in this work uses a piezoelectric transducer to evaporate ethanol at a constant rate (Figure 4b left). The intensity of this source can also be adjusted through the piezoelectric driver, but, in this work, it was kept constant at about 7 μg/s. All experiments can be defined, monitored, and logged through a custom software solution developed with Node.js (https://nodejs.org/) and running on an Orange Pi 3 single board computer (SBC) (Xunlong Software, Shenzhen, China), which guarantees all real-time communications and database storage through an InfluxDb (https://www.influxdata.com/). The interface with the system is made through a web-page and Socket.Io (https://socket.io/) communication.

### 5.2. Mobile Robot

A modified version of the mobile robot used by [20] was developed for these experiments (Figure 4b right). This is a two-wheeled, differential-driven unit with odometry, a Marvelmind v4.9 (Marvelmind Robotics, Tallinn, Estonia) beacon for global localisation and a 360° LiDAR for obstacle avoidance. The robot has 160 mm diameter and a height of 300 mm, when the localisation beacon is installed. It uses an STM32 MCU to support low level motion control and an ESP32 MCU for high-level navigation and Wi-Fi communication. Additionally, the robot contains a gas sensing unit and a 2D anemometer for environmental sensing. The navigation, data processing algorithms are implemented in C++ and Python as Robot Operating System (ROS) (https://www.ros.org/) nodes.

### 5.3. Odour Compass

The robot senses the environment with a metal oxide gas sensor (MOX) (three sensors were installed in the robot, but only one, at the same height as the gas source, was used in this work) and a custom 2D thermal anemometer. MOX sensors are resistive transducers that decrease their resistance in atmospheres containing oxidising vapours, such as ethanol. The sensor used in this work was a MiCS-5524 from SGX Sensortech (Corcelles-Cormondreche, Switzerland). This sensor can detect few parts per million (ppm) of ethanol vapour and has a rise time constant of about 1–2 s, which is relatively fast for chemical sensors, but a slower fall time constant of 5 to 10 s. The gas sensor was calibrated by a process like the one described in [21], using an enclosed box, where the concentration of ethanol was changed in a set of known values. For experiments involving multiple different gases, an array of MOX sensors, such as the one used in [22], could have been used.

The top of the robot contains a thermal anemometer composed by an air deflecting cylinder surrounded by five self-heated thermistors that measure the airflow intensity at their locations. The global airflow intensity around the robot and the airflow direction are estimated by processing the response of all five measuring elements, as described in [23].

These two sensing systems are sometimes referred to as an odour compass, by their ability to provide information to estimate the direction of an odour source, measuring the gas concentration c(x,y,t) and the airflow vector u(x,y,t) at a given position (x,y).

## 6. Experimental Results

While developing this work, the proposed method was tested and validated more than 50 times in the described environment, placing the source in different positions and using different airflow intensities. In these tests, the method was able to provide consistent estimates of the defined state vector, and, in particular, consistent estimates of the odour source localisation. This section summarises some of these results, characterising first the performance of the estimation process, while the robot is covering the environment, and later characterising that same performance, but for three different trajectories: one of the best trajectories obtained from the GA and two empirically devised trajectories. In order to keep the results comparable, the shown results were taken in the same environmental conditions: the odour source was placed at (0.5,0,0.2) and its intensity was set to a release rate *Q* of approximately 6 μg/s. The wind $\bar{u}$ was set to uniform at 0.31 m/s speed. The algorithm was developed in Python and optimized with the Numba JIT compiler. Using a PC equipped with an Intel I7-8750H CPU and 16 GB RAM, it takes roughly 120 ms to perform 60 iterations of the particle filter algorithm with 1000 particles. Disabling the Numba optimisation, the execution time rises to approximately 3 s.

### 6.1. Plume Intensity and Dispersion

**Figure 5:**
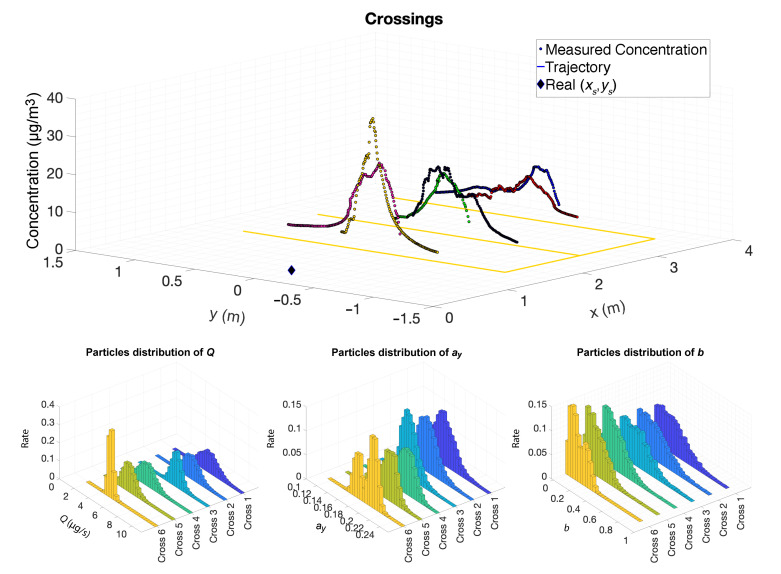
Trajectories used to evaluate the evolution of the estimation process while six crossings were performed at three downwind distances. The top figure shows the trajectory and the concentration measurements along the experiment. The particles distribution is shown in the bottom figures.

The current work estimates five parameters from a Gaussian plume. In order to evaluate how the estimation process evolved for the intensity and the dispersion, an experiment was designed consisting of crossing the plume twice, in the opposite direction, at three downwind distances from the source (1, 2 and 3 m), as shown in Figure 5 (top). The robot was moved slowly, at a speed of 20 mm/s. The concentration measurements show that the plume dispersion increases with the distance to the chemical source, as predicted by the Gaussian Plume model. Despite the relatively slow motion, it is possible to observe a short response time, when entering the active region of the plume, and a longer recovery time when leaving the plume, caused by the slower falling dynamics of the gas sensor, which insert distortions in the spatial measurements and cause errors in the estimation process. The intermittent and chaotic nature of the plume is also observed by the rapid changes on the instantaneous concentration values along the trajectories, resulting in a rugged concentration curve, compared to the smooth average values provided by the Gaussian Plume model.

The distribution of the estimates of the multiple parameters can be estimated by the distribution of the values of those parameters contained in the population of particles. The evolution of those distributions is shown in Figure 5 (bottom) for the parameters *Q*, $a_{y}$, and *b*. This analysis was done by fixing the parameters $x_{s}$ and $y_{s}$ equal to their true value and leaving the other parameters free to be adjusted by the filter. It is clearly visible that the variance of all parameters reduces while the robot increasingly covers the plume and approaches the source, and it is also possible to see that the average of *Q* tends to the true value and the horizontal dispersion coefficients are coherent with what would be expected for a laminar atmosphere (see [13]).

### 6.2. Sampling Strategy Analysis

In order to validate a cover strategy proposed by the GA, the odour plume was covered by three different trajectories: one meeting the criteria proposed by the GA for a good coverage and two other trajectories with different angles. Table 3 shows the angles and number of crossings of each trajectory. The first and third trajectories were devised based on empirical experimentation, whereas the second one was optimised by the GA.

As the wind tunnel’s length and width is 10 times smaller than the simulated environment, the threshold for acceptable estimation errors is set to 0.5 m. Each trajectory was performed five times under the same conditions.

To evaluate whether there are statistically significant differences in the obtained results, 100 independent estimation trials were performed for each trajectory, and the Wilcoxon test was employed to compare the data, with a 95% confidence interval. The results of this test, presented in Table 4, show that at the chosen confidence level there are statistically significant differences between the estimations obtained from all trajectories.

The impact of the number of particles on the quality of the estimation was also evaluated and presented in Table 5. This table shows that the improvement in the estimation process for 500 and 5000 particles was minimal, which may be explained by the reduction of the search space provided by the prior estimation.

**Figure 6:**
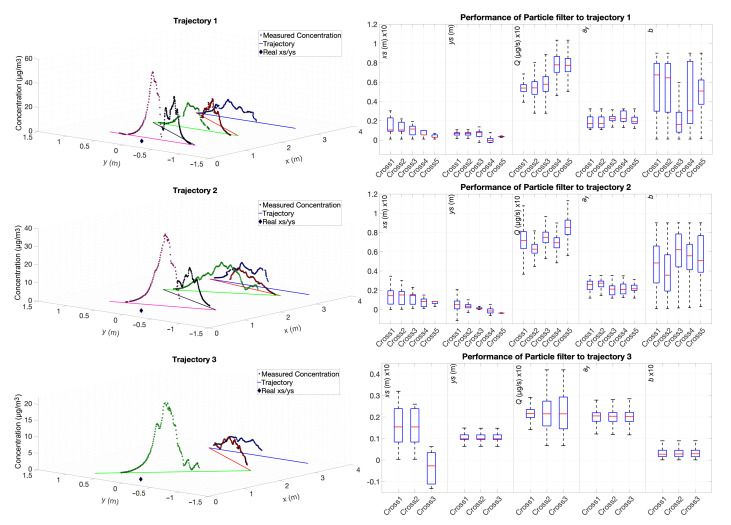
Dataset of three trajectories with different angles. Trajectory 1 (top row) was performed with angles 0°, 45°, 0°, 45°, and 0° relatively to crosswind. Trajectory 2 (middle row) was evaluated with 0°, 20°, 43°, 40°, and 39°, respectively, and the trajectory 3 (bottom row) with angles 0°, 47°, and 63°. The left column shows the trajectories, measured concentrations along the plume crossings, and the real odour source location. The right column shows the performance of the algorithm along the crossings. Some of the boxplots were scaled by 0.1 to improve the visualisation of the data.

**Figure 7:**
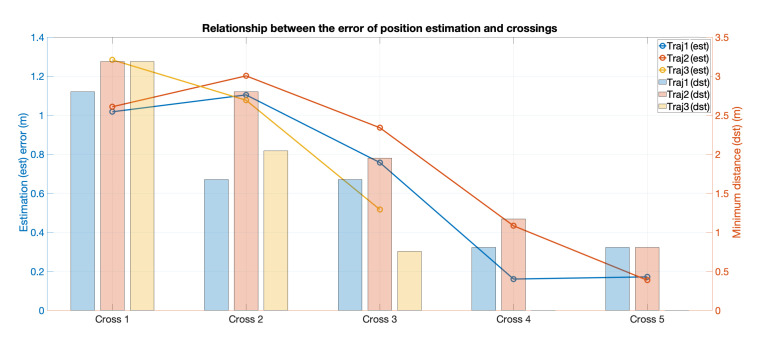
Evolution of the estimation performance of each trajectory along the plume crossings. The dot points show the location estimation error according to the crossings. The bar plot shows the minimum Euclidean distance to the odour source.

Figure 6 (left) shows an example run of the measured concentration values during each motion where the active region of the plume is detected, and the estimation results for each crossing are also presented in this figure (right). Figure 7 presents the results of the three trajectories where the evolution of the estimation error along with the respective distance to the source is shown. Trajectory 1 and trajectory 2 show better estimation results than trajectory 3. The same figure shows that, with fewer crossings (larger angles), the estimation error of the position increases at the same Euclidean distance to the source. All three trajectories have acceptable estimation results. The parameter $x_{s}$ is the most sensitive in the whole process. It contains a large uncertainty in the beginning of the process, but, as the robot crosses the plume and moves closer to the source, its error and uncertainty becomes smaller, until about 10 cm. The centre of the plume $y_{s}$ is always accurately estimated, from the beginning of the process. We do not have a ground truth for the dispersion coefficients, but the values obtained for parameters $a_{y}$ and *b* are in line with what would be expected for a very stable environment, such as the one we employed in the experiments. The emission rate *Q*, together with $x_{s}$, are the most unstable and uncertain parameters, needing more plume crossings to obtain more confident estimates. This is attributed to the variability of the instantaneous concentration measurements that may be far from the expected value and highly influence the estimate of those two parameters. The dynamics of the environment along with the response of the sensor can have a significant impact on the readings that, when compared to the hypothesis evaluated with the Gaussian Plume, resulting in plumes with different widths and emission rates, increasing the error on the estimations. Figure 6 shows that trajectories with angles larger than 45° increase the uncertainty of the estimates, with particular impact on the estimate of *Q*. The estimation process seems to not be highly affected by the crossing angle, when the robot is moving close to the odour source.

## 7. Conclusions

The quality of a Source Term Estimation process depends both on the quality of the estimation algorithm and on the quality of the data used in the estimation. This work proposes a GA-based approach that generates optimal trajectories to guide a mobile robot across the active region of an odour plume in order to sample valuable data to be used by a particle filter-based algorithm used in the STE of the plume. Using trajectories with crossing angles in the range from 12° to 45° tends to generate better estimates, reducing the uncertainty of the results. The proposed implementation efficiently estimates a five-dimensional state vector by a two-step approach: the initialization of hypothesis from a limited search space defined by conventional inverse processes, and a later refinement of this hypothesis through a particle filter that runs after each plume crossing with a robot. The methodology was extensively tested and validated in a controlled environment, having systematically provided good estimation results. This controlled environment allows for keeping constant and predictable conditions, which are key to comparing results in this area. In the future, the proposed method shall be tested on plumes with higher disturbance and further developed to estimate their three dimensions, exploring the vertical dispersion of the odour.

## Figures and Tables

**Table 1:** Parameters of the genetic algorithm.

Parameter	Value	Description
*gens*	100	Number of generations used
*pop_size*	100	Size of the population used by the algorithm
*elite_size*	10	Amount of individuals from the previous population that survive into the next
*n_offspring*	50	Number of offspring created per generation
*domain*	(0, 0.7×π/2)	Interval of possible offsets
*p_cross*	0.7	Crossover rate
*p_mut*	0.3	Mutation rate
σ	10% domain amplitude	Standard deviation of the Gaussian mutation
*tourn_size*	3	Size of the tournaments for selecting the parent individuals

**Table 2:** Parameters of the simulator.

Parameter	Value	Description
$W_{s}$	0.05 m/s	Initial wind speed
$W_{d}$	0 rad	Initial wind direction
$W_{v}$	0.01 rad	Standard deviation of the Gaussian noise added to the wind vectors
$F_{r}$	0.1 Hz	Filament emission rate
γ	0.05 m/s	Filament growth rate
*Kx*	5	Parameter of the wind equations
$D_{arena}$	40 m × 30 m	Dimensions of the arena
Cellsize	7.2 m	Width of the cells for computing the wind
$S_{region}$	(38 m, 21 m)	Start region of the robot
$S_{step}$	0.5 s	Duration of the simulation step
$S_{time}$	1800 s	Maximum evaluation time

**Table 3:** Evaluated trajectories and respective angles.

Trajectory	$1^{{}^{\circ}}$ Cross Angle	$2^{{}^{\circ}}$ Cross Angle	$3^{{}^{\circ}}$ Cross Angle	$4^{{}^{\circ}}$ Cross Angle	$5^{{}^{\circ}}$ Cross Angle
1	$0^{{}^{\circ}}$	$45^{{}^{\circ}}$	$0^{{}^{\circ}}$	$45^{{}^{\circ}}$	$0^{{}^{\circ}}$
2	$0^{{}^{\circ}}$	$20^{{}^{\circ}}$	$43^{{}^{\circ}}$	$40^{{}^{\circ}}$	$39^{{}^{\circ}}$
3	$0^{{}^{\circ}}$	$47^{{}^{\circ}}$	$63^{{}^{\circ}}$	−	−

**Table 4:** Results of the Wilcoxon test (Z|p).

State	Traj1-Traj2	Traj1-Traj3	Traj2-Traj3
$x_{s}$	9218|0.0	11,503|0.0	860|0.0
$y_{s}$	3819|0.0	25,006|0.0	14,714|0.0
*Q*	25,204|0.0	0|0.0	0|0.0

**Table 5:** Mean and standard deviation results from multiple runs with different amounts of particles.

N. Particles	$x_{s}$ (m)	$y_{s}$ (m)	*Q* (μg/s)	$a_{y}$	*b*
100	0.39±0.2262	0.05±0.0620	7.44±2.4267	0.22±0.0518	0.38±0.1852
500	0.40±0.2096	0.06±0.0562	7.51±2.2206	0.22±0.0441	0.38±0.1655
5000	0.38±0.2028	0.06±0.0560	7.25±2.0291	0.21±0.0359	0.40±0.1558

## Abbreviations

The following abbreviations are used in this manuscript:

GA	Genetic Algorithm
GP	Genetic Programming
MOX	Metal Oxide Gas Sensor
PDF	Probability Density Function
SIR	Sequential Importance Resampling
STE	Source Term Estimation
